# EHPnet: Healthy House Institute

**Published:** 2008-01

**Authors:** Erin E. Dooley

The mission of the Boise, Idaho–based Healthy House Institute (HHI) is to educate homeowners on ways to make and keep their home environments as healthful as possible. Toward this end, the HHI has compiled data in areas including indoor air, drinking water, building and remodeling, housekeeping, and energy efficiency on its website, **http://www.healthyhouseinstitute.com/**.

Information is presented in seven topic categories: Air, Building, Design, Energy, Health & Safety, Materials & Furnishings, and Water. Each topic page presents resources organized by four information types: Reviews; Tools; Articles, Hints, & Tips; and Books & Videos. The Reviews category links to overviews of products inspected by HHI that perform well in such areas as safety, energy use, allergen reduction, and use of chemicals. The Tools category lists products that can be used in building or remodeling to make a healthier home. These include paints and coatings, lighting fixtures, indoor air cleaners, and pest control strategies.

The Articles, Hints, & Tips section provides information on new green products, how to choose sustainably produced building materials, green roofs, and energy efficiency. The Books & Videos page lists educational materials such as *Green Building A to Z* (an introduction to sustainable housing), *Green Clean* (a primer on eco-friendly housekeeping), and *The Mold Survival Guide* (which offers advice on eradicating mold while minimizing health risks). Visitors can also consult the “HHI-pedia,” a list of definitions for terms used in green building and housekeeping.

